# Recent advancements on the use of 2-methyltetrahydrofuran in organometallic chemistry

**DOI:** 10.1007/s00706-016-1879-3

**Published:** 2016-12-07

**Authors:** Serena Monticelli, Laura Castoldi, Irene Murgia, Raffaele Senatore, Eugenia Mazzeo, Judith Wackerlig, Ernst Urban, Thierry Langer, Vittorio Pace

**Affiliations:** Department of Pharmaceutical Chemistry, University of Vienna, Vienna, Austria

**Keywords:** Solvent effect, Carbanions, Grignard reaction, Green chemistry

## Abstract

**Abstract:**

Since the introduction of 2-methyltetrahydrofuran as an useful alternative to the classical tetrahydrofuran, there has been a continuous interest in the synthetic community operating at academic and industrial towards it. In particular, the much higher stability that basic organometallic reagents display in 2-methyltetrahydrofuran makes it suitable for processes involving such sensitive species including asymmetric transformations. The easy formation of an azeotropic mixture with water, the substantial immiscibility with water, and the fact it derives from natural sources (corncobs or bagasse), allow to consider it in agreement with the Anastas’ Geen Chemistry principles. In this minireview, selected examples of its employment in organometallic transformations ranging from carbanions to radical and transition metal-catalyzed processes are provided.

**Graphical abstract:**

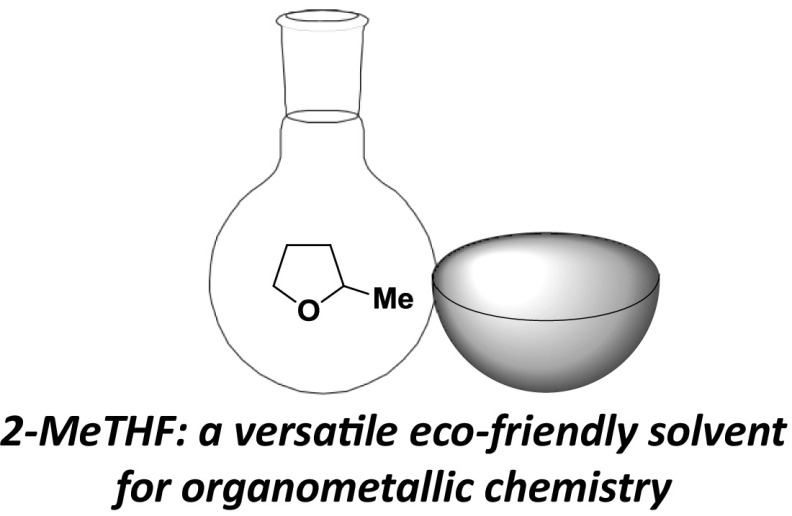

## Introduction

Solvents featuring ether functionalities constitute highly valuable media for performing chemistry with organometallic reagents [1]. This is mainly due to their capability to disaggregate such species, thus modulating their reactivity [2]. Unfortunately, common solvents such as tetrahydrofuran (THF) tend to react with highly basic carbanions, thus requiring the employment of low temperatures to suppress undesired side reactions [3–5]. In this context, it is quite known since seminal studies by Bates that THF undergoes an extremely fast lithiation in the presence of *n*-BuLi at the C-2 (*t*_1/2_ = 10 min at 35 °C), followed by a reverse [3 + 2] cycloaddition, giving ethylene and acetaldehyde [6]. On the other hand, the simple presence of a methyl group at the 2-position of 2-methyltetrahydrofuran (2-MeTHF) has a dramatic effect on the decomposition, as reflected by the much higher value of *t*_1/2_ = 130 min at 35 °C) (Scheme 1).

**Figure:**
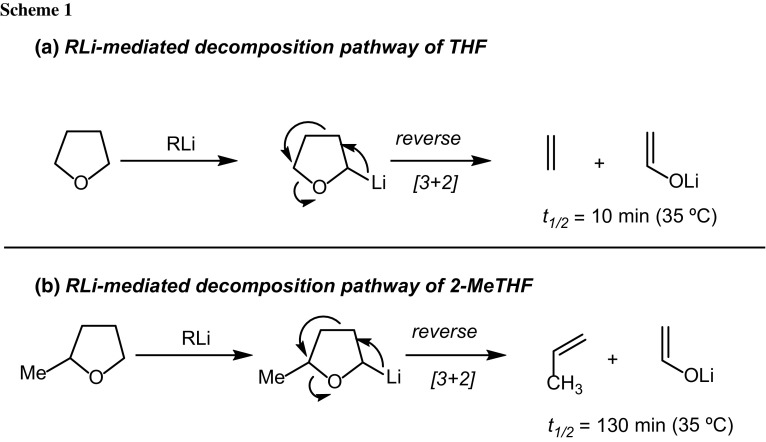

Solutions of organometallic reagents in 2-MeTHF [7–9] feature higher stability and solubility compared to those ones in THF [10]; this concept maybe extended also to hydrocarbon solvents in which often solution of organolithiums are supplied. For example, the presence of 2–2.5 mol amounts of 2-MeTHF per mole of MeLi increases the stability of MeLi in cumene, thus avoiding the need of Me_2_Mg as a stabilizer; further stabilization is achieved by the addition of LiBr [11]. In addition to the greater thermal stability, 2-MeTHF may also provide a supplementary advantage for a MeLi/MeTHF/cumene compositions. In fact, the storage of MeLi/THF/cumene below 0 °C eventually forms (MeLi/THF)_4_ crystals that can be easily redissolved with agitation and warming to room temperature. Similarly, crystallization during cold storage was also observed with benzyllithium in THF, but when 2-MeTHF was used instead of THF, the formation of benzyllithium-MeTHF solids was considerably decreased, and the thermal stability significantly increased. Apparently, 2-MeTHF provides enough spatially arranged variations of solvated aggregates, and thus, decreases the possibility for crystallization to take place.

Additional reasons motivate the significant use of 2-MeTHF in organic synthesis as a versatile and effective alternative to THF [7]: (a) the only partial miscibility with water (14/100 g) accounts for clean and easy work-up procedures, thus dramatically decreasing the need of classical organic solvents for extracting the reaction products; (b) a standard distillation, not requiring dangerous dehydrating agents, provides dry solvent suitable for performing sensitive organometallic chemistry; (c) the high boiling point (82 °C) allows to run processes at higher temperatures with contemporaneous decrease of reaction times; (d) toxicological essays excluded the risk of genotoxicity and mutagenicity during the exposure to this solvent [12]; (e) because of the low dielectric constant it possesses (*ε* = 6.97) [9], its physical properties resemble also those ones of toluene, thus expanding the range of solvents it can replace. Moreover, it could be obtained through the catalytic reduction of furfural and levulinic acid which are themselves available by dehydration of C-5 sugars present in biomass [13], thus in agreement with the seventh principle of Green Chemistry [14–19]. Unfortunately, the formation of peroxides cannot be avoided when employing this solvent in analogy to THF; however, the use of stabilizers modulate positively this drawback [9].

The aim of this minireview is focussing on recent applications (appeared in the last 5 years) of this solvent in organometallic reactions ranging from classical carbanionic to cross-coupling processes [17, 20–56].

## Use of 2-MeTHF in reactions involving carbanionic and nucleophilic species

Azzena reported that the generation and reactivity of single-electron transfer reagents such as 1,2-diaryl-1,2-disodioethanes is best accomplished in 2-MeTHF in alternative to THF [57]. Solution of this dianion in 2-MeTHF proved to be stable under dry Ar in a refrigerator for at least 24 h. Their results suggest that the employment of 2-MeTHF as a solvent promotes the nucleophilic substitution pathway of 1,2-diaryl-1,2-disodioethanes with 1,3-dichloropropane to a higher extent. Interestingly, significant differences were observed in the behavior of these organometals in 2-MeTHF and in cyclopentyl methyl ether (CPME) [58]. While comparable result were observed in a series of redox reactions, (e.g. reductive deprotection of the 2-bromoethyl ester of benzoic acid and reductive dechlorination of acids) 2-MeTHF proved to be the solvent of choice in reactions involving these dianionic species as nucleophiles or, even more dramatically, as bases. In the case of arylacetic acids, the *vic*-dianons perform a selective deprotonation in 2-MeTHF at the benzylic position, enabling the functionalization through subsequent treatment with electrophiles (E). Significantly, under analogous conditions halogenated aryloxy acetic acid derivatives undergo reductive dechlorination. Such an effect of the reaction medium can be rationalized by assuming that the interaction between the solvent and the diorganometals strongly influence the reactivity of these intermediates, either by affecting their aggregation states and/or reaction products (Scheme 2).

**Figure:**
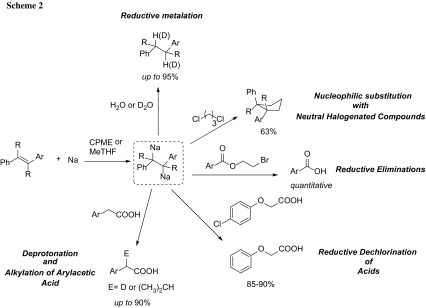

Breit and coworkers developed a general method for the preparation of primary and secondary alkylmagnesium reagents starting from alkenes (via hydroboration) through the boron-magnesium exchange on alkyl boronates in 2-MeTHF [59]. The authors demonstrated the synthetic usefulness in a wide range of carbon–carbon bond forming reactions, including iron, palladium, and copper-catalyzed cross-couplings. Synthetically useful methallyl alcohol- and homomethallyl alcohol-derived borolanes equipped with typical silicon-based protecting groups are highly sensitive substrates and required the slow addition of the dimagnesium reagent at 0 °C followed by the slow warming to ambient temperature to avoid side reactions, being 2-MeTHF the best solvent (Scheme 3).

**Figure:**
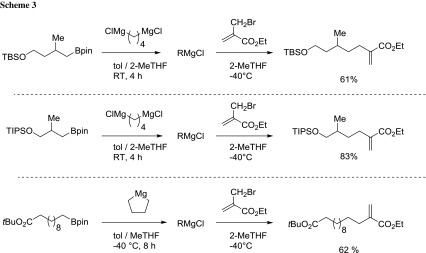

Luisi et al. performed a direct and sustainable synthesis of tertiary butyl esters by the addition of organolithiums to (Boc)_2_O under microfluidic conditions by employing 2-MeTHF as the solvent [60]. The reactions conducted under batch condition needed cryogenic temperatures, and considerable amounts of the corresponding tertiary alcohols were formed as a consequence of the predictable multiple addictions. By switching to the corresponding flow technique a more efficient, versatile and selective transformation was achieved. This protocol worked well in the case of several aryl and heteroaryl bromides, different acetylene and was also extended to β-bromostyrene (using *s*-BuLi as a lithiating agent) (Scheme 4).

**Figure:**
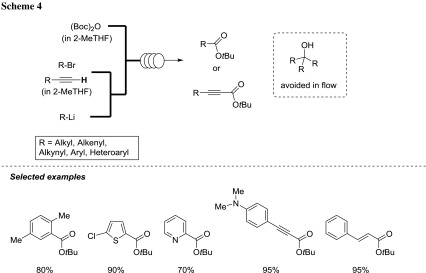

The same Luisi’s group exploited the combination of using, in flow technology, 2-MeTHF for the synthesis of enantioenriched alcohols via the Corey–Bakshi–Shibata (CBS) oxazaborolidine-mediated reduction of the prochiral ketones [61]. Under the optimized reaction conditions, the process reached to completion within few minutes, thus providing the desired asymmetric targets in up to 99% yield and 91:9 enantiomeric ratio (*er*) (Scheme 5).

**Figure:**
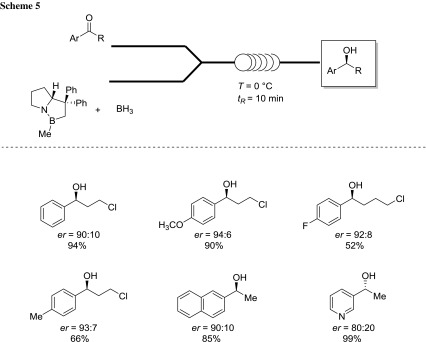

Fjelbye et al. prepared a chiral 1,3-amino alcohol (as hydrochloride salt) using a two-step approach involving the magnesiation of 2-iodopyridine in 2-MeTHF followed by the reaction with the chiral sulfinamide direct precursor of the desired enantiopure compound [62]. The following points merit mention: (a) the use of the Turbo Grignard reagent (*i*-PrMgCl LiCl) guarantees the avoiding of homocoupled products; (b) 2-MeTHF is the optimal solvent in terms of both reaction yield and, more importantly, diastereoselectivity (Scheme 6).

**Figure:**
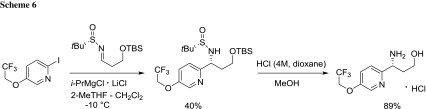

Ronn’s group described the regioselective deprotonation at C-2 of 3-bromofuran with LDA followed by the DMF-mediated formylation [63]. 2-MeTHF gave the best results in terms of purity and yield also considering the simple and straightforward work-up procedure it allowed. The method enabled to produce the desired compound in multi hundred gram batches with an overall yield of 85–95% yield (Scheme 7).

**Figure:**
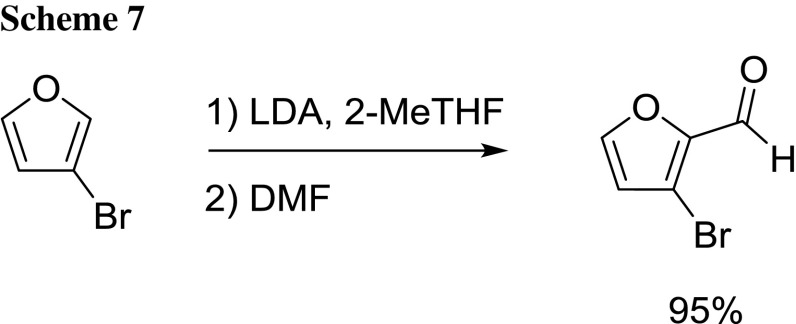

Grellepois described the development of a short efficient, and general synthesis of enantiopure β-(trifluoromethyl) β-amino acids containing a quaternary stereogenic center at the β position [64]. It is reported the use of the Reformatsky reagent in the synthesis (on large scale), of various enantiopure *N*-*tert*-butanesulfinyl trifluoromethyl β-amino esters from bench-stable analogues of aliphatic and aromatic trifluoromethyl *N*-tert-butanesulfinyl ketoimines. Optimization studies pointed out that 2-MeTHF was the best solvent while, decreasing the temperature to 0 °C plays a beneficial effect on diastereoselectivity and yield (Scheme 8).

**Figure:**
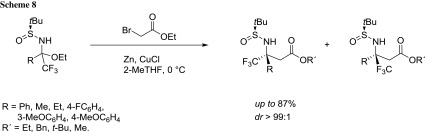

Schmalz’s group developed new Tartrol-derived chiral phosphine-phosphite ligands (L1) to perform enantioselective Cu-catalyzed 1,4-addition reactions of Grignard reagents to cyclohexenone in 2-MeTHF with very high enantio- and regio-selectivities (Scheme 9) [65]. In the best case, the 1,4 addition product was obtained in 84% *ee* and 85:15 regioselectivity, using EtMgBr as reagent under standard reaction conditions (4% mol of CuBr–SMe_2_, 6 mol% of L1, 2-MeTHF, −78 °C and slow addition of the Grignard reagent).

**Figure:**
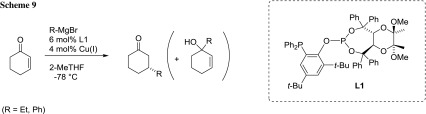

Pace and coworkers developed a highly chemoselective protocol for transforming iso(thio)cyanates into the corresponding (thio)amides [66–70]; in particular, the treatment of isocyanates with the in situ generated Schwartz reagent [71] provides a smooth access to formamides (Scheme 10) [72]. Both the selection of 2-MeTHF and this hydride source proved to be pivotal for obtaining high chemocontrol on multifunctionalized isocyanates.

**Figure:**
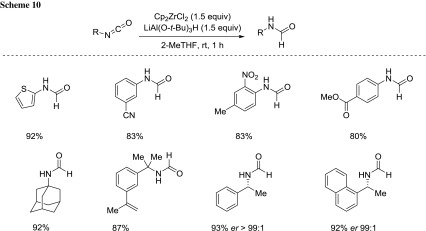

Nardi and coworkers reported a chemoselective version of the classical Luche reduction of α, β-unsaturated carbonyl compounds in the presence of substoichiometric amount of sodium borohydride under erbium triflate catalytic conditions in 2-MeTHF [73]. Under the optimal conditions [i.e., Er(OTf)_3_ (5 mol%), NaBH_4_ (0.75 equiv)], the desired allylic alcohols are obtained in selectivities up to 96:4 (Scheme 11).

**Figure:**
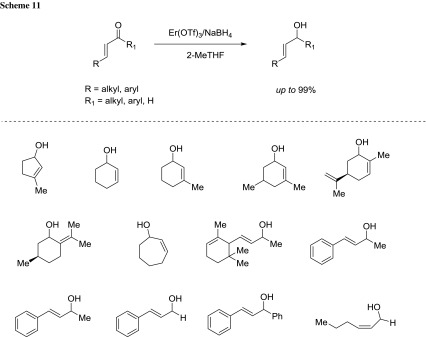

Maudit’s group developed a new family of phosphine ligands (DIPPAM) (L2), which promoted a very efficient Cu-catalyzed 1,4 addition of dialkylzinc to both cyclic and acyclic enones. The methodology could be adequately adapted to the analogous 1,6-conjugate addition of dialkylzinc to cyclic dienones using 2-MeTHF as solvent at 0 °C [74] (Scheme 12).

**Figure:**
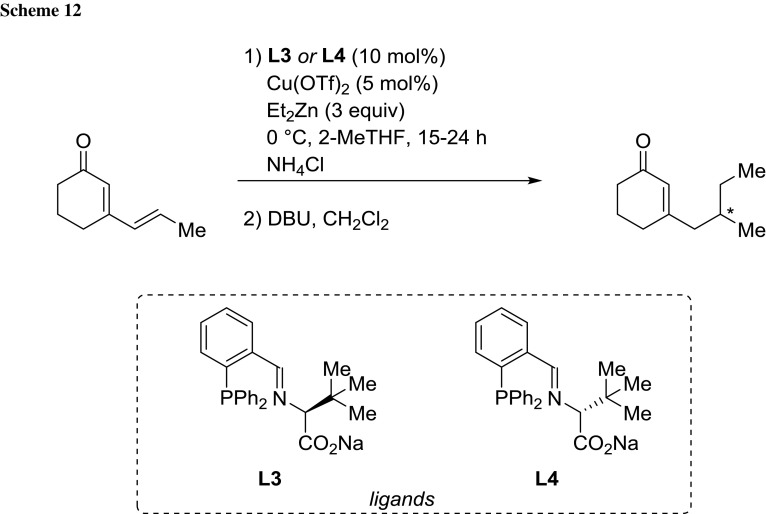

## Use of 2-MeTHF in transition metal catalyzed chemistry

Mondal et al. reported the application of 2-MeTHF in a Pd-catalyzed Suzuki type carbonylation reaction via the cleavage of the C–Cl bond of acid chlorides to yield aryl ketones (Scheme 13) [75]. The model reaction between benzoyl chloride and phenylboronic acid clearly evidenced the superiority of this solvent compared to different ones. Moreover, using 2-MeTHF the crude mixture of the cross-coupling product was simply extracted by quenching with water, followed by the separation of the resulting 2-MeTHF/water phases and drying without the need to use additional organic solvent during the whole work-up procedure.

**Figure:**
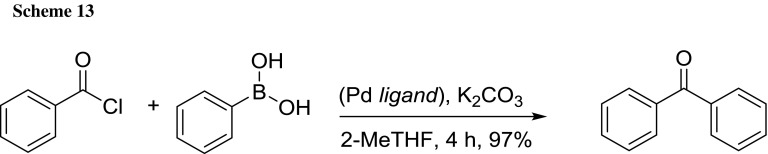

Garg documented the nickel-catalyzed Suzuki–Miyaura cross-coupling between aryl halides and (hetero)aromatic boronic acids in 2-MeTHF (Scheme 14) [76]. The scope of the reaction is broad with respect to both coupling partners and, the possibility to work at gram scale using low catalyst loadings renders the protocol highly attractive for industrial applications.

**Figure:**
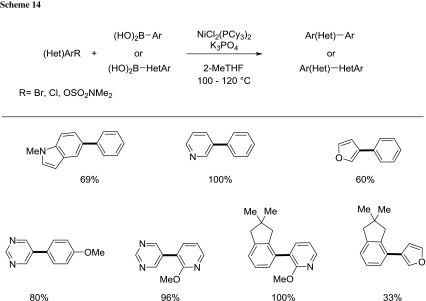

Furthermore, they extended the procedure to the efficient formation of aryl C–N bonds under analogous nickel catalysis conditions, thus providing an efficient access to aryl amines in synthetically useful yields [77] (Scheme 15).

**Figure:**
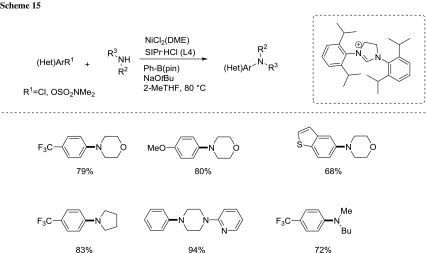

Levahcer et al. described in 2015 the Pd-catalyzed carbonylation of (hetero)aryl, alkenyl, and alkyl halides with *N*-hydroxysuccinimidyl formate as CO surrogate [78]. A large range of aryl, vinyl, allyl, and benzyl halides can be transformed into the corresponding NHS esters in good to excellent yields under mild conditions (60 °C/10 h/2-MeTHF; Scheme 16).

**Figure:**
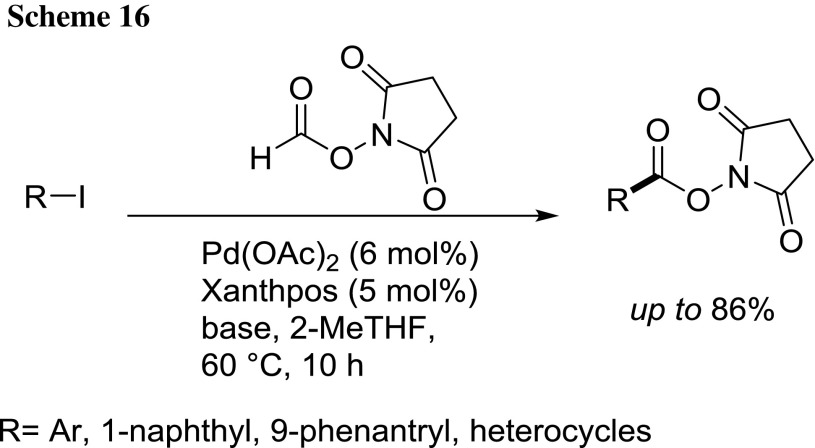

Maes et al. developed a novel palladium-catalyzed aerobic oxidation to access guanidine-containing and related heterocycles from bisnucleophiles and aliphatic isocyanides in 2-MeTHF [79] (Scheme 17). The protocol is highly versatile, thus enabling a fast access to a plethora of pharmaceutically relevant heterocyclic systems (e.g., astemizole and norastemizole). The simplicity of the experimental procedure, the use of bench (i.e., non distilled 2-MeTHF), the low catalyst loading and atmospheric pressure render the overall method environmentally benign.

**Figure:**
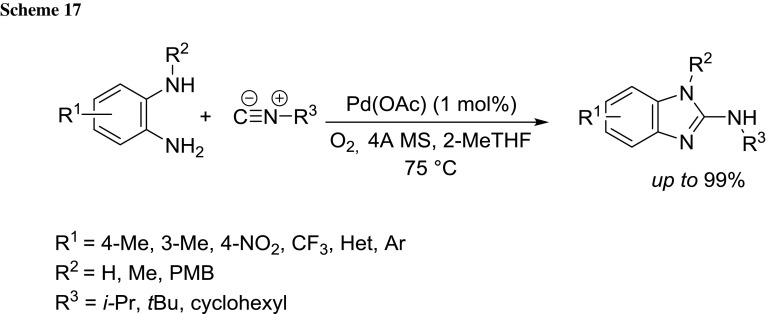

In 2013 Procter developed the first asymmetric silylation of unsatured lactams and amides using a Cu(I)-NHC catalyst and PhMe_2_SiBpin as silyl donor (Scheme 18) [80]. In the study, 2-MeTHF proved to be a very attractive alternative to THF in terms of both reaction yield and enantioselectivity. The methodology was applied to the synthesis of the (*R*)-enantiomer of the nootropic drug oxiracetam.

**Figure:**
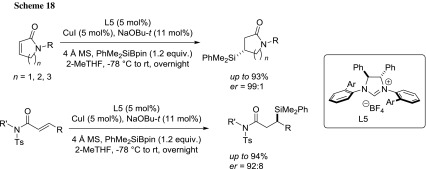

Carreira’s group reported the first, direct enantioselective iridium catalyzed substitution of racemic secondary allylic alcohols with sulfamic acid in 2-MeTHF to yield optically pure amines (Scheme 19) [81]. The method tolerates a wide range of allylic alcohols (aliphatic, aromatic, heterocyclic) and gives the corresponding amines with very good enantioselectivities (up to 99%).

**Figure:**
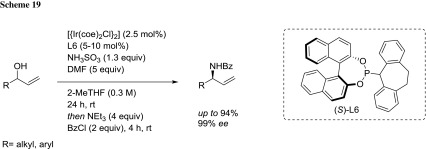

Frost et al. reported a ruthenium-catalyzed ortho C–H alkenylation of a wide range of *N*-aryloxazolidinone analogues in 2-MeTHF [82]. The reaction proceeded with complete monoselectivity, as indicated by >27 examples (Scheme 20).

**Figure:**
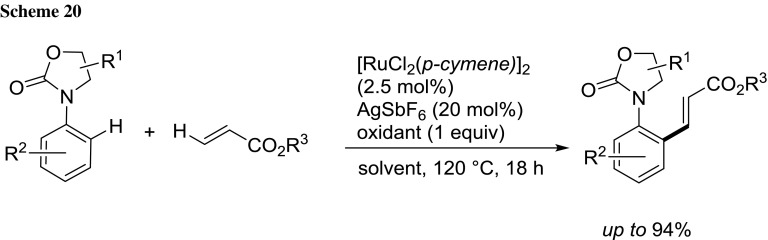

## Conclusions

The use of 2-MeTHF as a solvent in synthetic chemistry has constantly raised in the last years: indeed, the beneficial properties it displays makes it a versatile alternative to the commonly employed THF. The additional presence of a methyl group at the 2-position profoundly increases the chemical stability towards highly basic organoalkali reagents, thus allowing to run reactions at higher temperatures and limiting the use of noxious and flammable solvents such as diethyl ether. From a practical perspective its limited miscibility with water allows to obtain a dry solvent for organometallic chemistry through a standard distillation. Additionally, it should be considered that work-up procedures do not need extraction processes by means of standard solvents (e.g., halomethanes, esters, or ethers). Taken together these properties with the adherence of its employment to the Green Chemistry principles, it is foreseen more and more applications of this solvent in organic processes both at laboratory and pilot scale.
